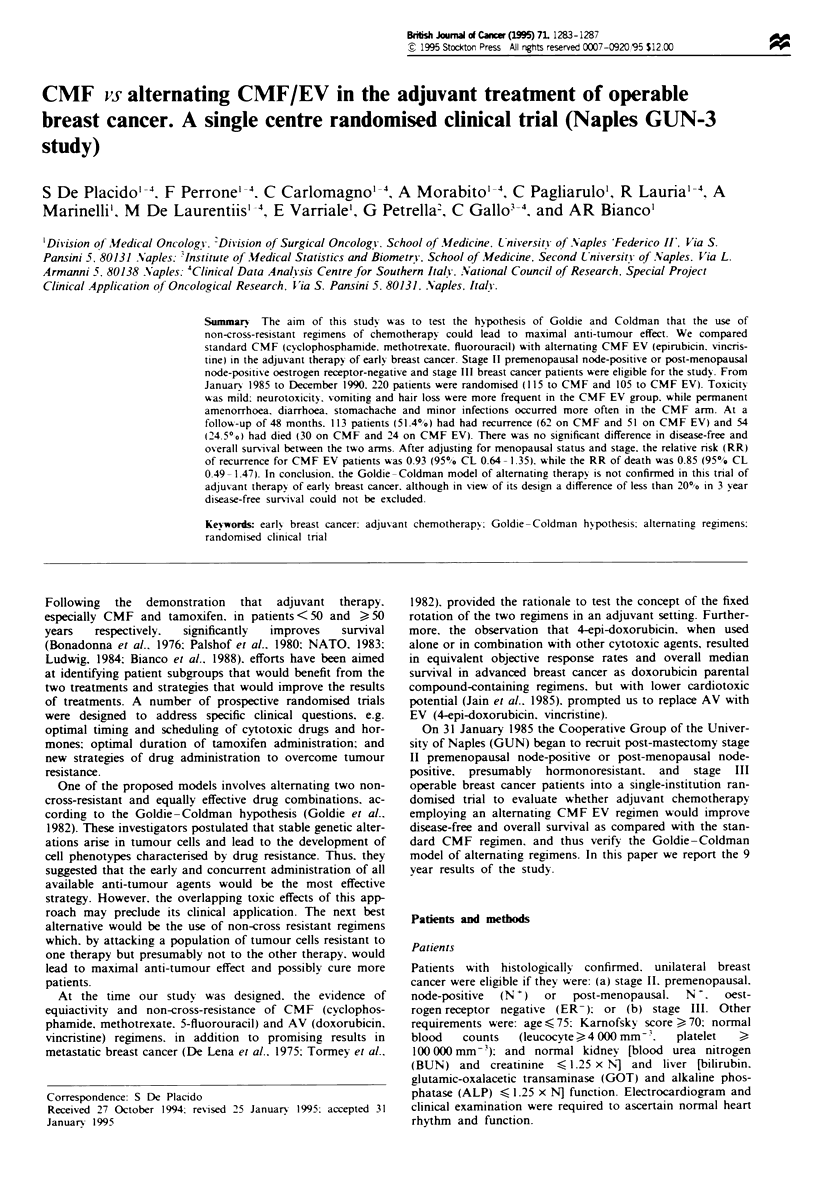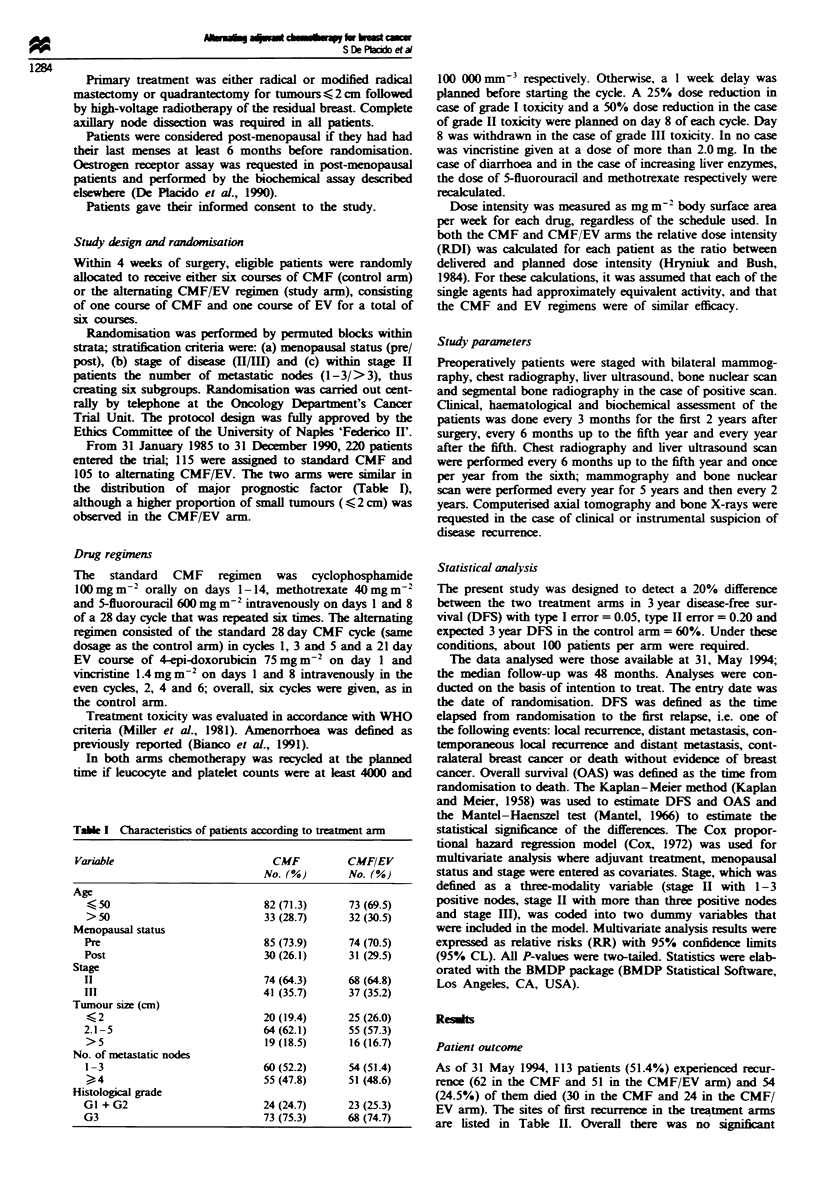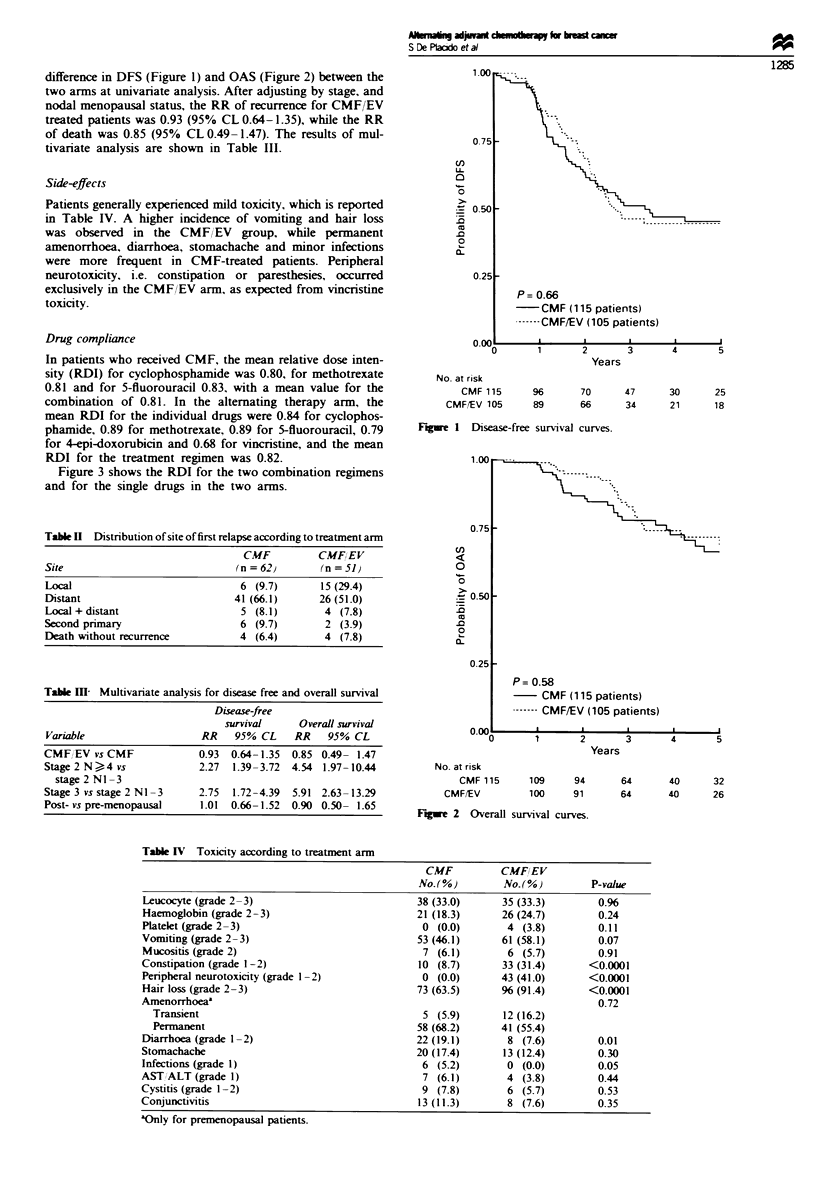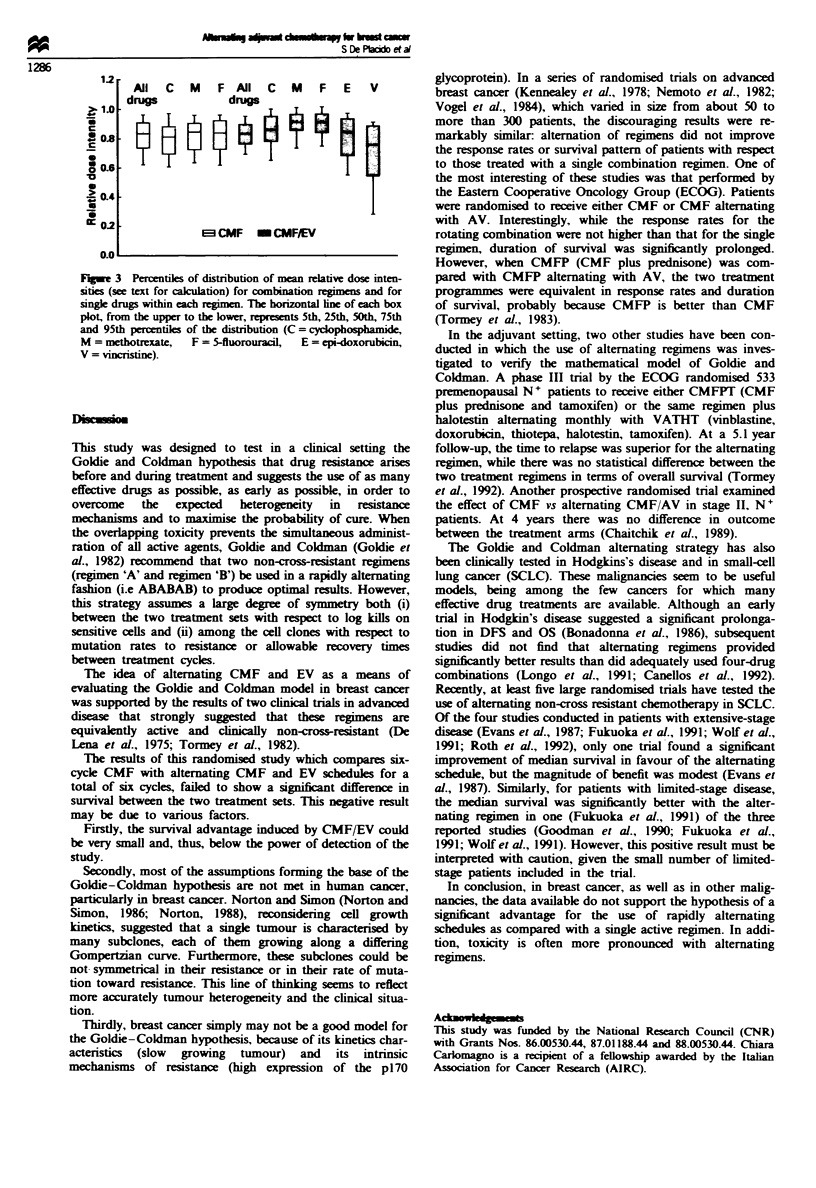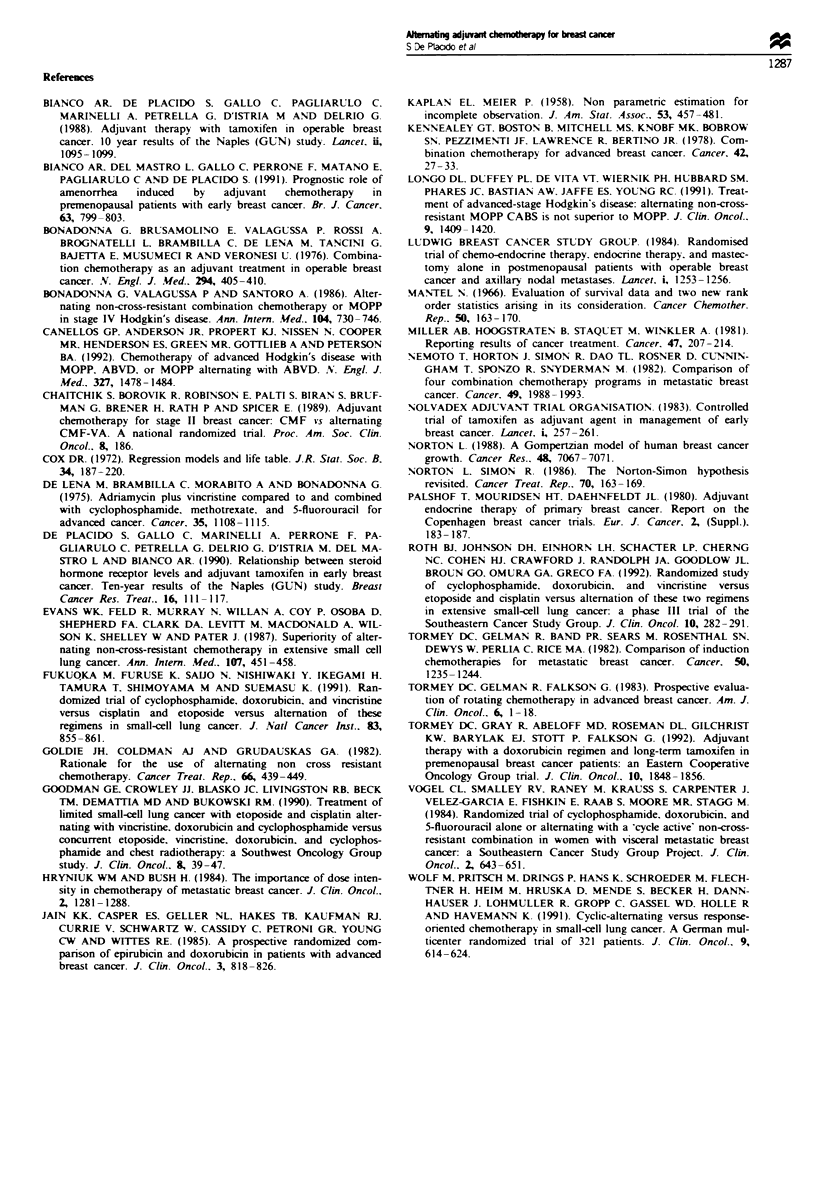# CMF vs alternating CMF/EV in the adjuvant treatment of operable breast cancer. A single centre randomised clinical trial (Naples GUN-3 study).

**DOI:** 10.1038/bjc.1995.248

**Published:** 1995-06

**Authors:** S. De Placido, F. Perrone, C. Carlomagno, A. Morabito, C. Pagliarulo, R. Lauria, A. Marinelli, M. De Laurentiis, E. Varriale, G. Petrella

**Affiliations:** Division of Medical Oncology, School of Medicine, University of Naples Federico II, Italy.

## Abstract

The aim of this study was to test the hypothesis of Goldie and Coldman that the use of non-cross-resistant regimens of chemotherapy could lead to maximal anti-tumour effect. We compared standard CMF (cyclophosphamide, methotrexate, fluorouracil) with alternating CMF/EV (epirubicin, vincristine) in the adjuvant therapy of early breast cancer. Stage II premenopausal node-positive or post-menopausal node-positive oestrogen receptor-negative and stage III breast cancer patients were eligible for the study. From January 1985 to December 1990, 220 patients were randomised (115 to CMF and 105 to CMF/EV). Toxicity was mild; neurotoxicity, vomiting and hair loss were more frequent in the CMF/EV group, while permanent amenorrhoea, diarrhoea, stomach ache and minor infections occurred more often in the CMF arm. At a follow-up of 48 months, 113 patients (51.4%) had had recurrence (62 on CMF and 51 on CMF/EV) and 54 (24.5%) had died (30 on CMF and 24 on CMF/EV). There was no significant difference in disease-free and overall survival between the two arms. After adjusting for menopausal status and stage, the relative risk (RR) of recurrence for CMF/EV patients was 0.93 (95% CL 0.64-1.35), while the RR of death was 0.85 (95% CL 0.49-1.47). In conclusion, the Goldie-Coldman model of alternating therapy is not confirmed in this trial of adjuvant therapy of early breast cancer, although in view of its design a difference of less than 20% in 3 year disease-free survival could not be excluded.


British Journal of Cancer (1995) 71, 1283-1287

© 1995 Stockton Press All rights reserved 0007-0920/95 $12.00

### CMF *vs* alternating CMF/EV in the adjuvant treatment of operable breast cancer. A single centre randomised clinical trial (Naples GUN-3 study)

S De Placido[1-4], F Perrone[1-4], C Carlomagno[1-4], A Morabito[1-4], C Pagliarulo[1], R Lauria[1-4], A Marinelli[1], M De Laurentiis[1-4], E Varriale[1], G Petrella[2], C Gallo[3-4], and AR Bianco[1]

[1]*Division of Medical Oncology*, [2]*Division of Surgical Oncology, School of Medicine, University of Naples 'Federico II', Via S. Pansini 5, 80131 Naples;* [3]*Institute of Medical Statistics and Biometry, School of Medicine, Second University of Naples, Via L. Armanni 5, 80138 Naples;* [4]*Clinical Data Analysis Centre for Southern Italy, National Council of Research, Special Project Clinical Application of Oncological Research, Via S. Pansini 5, 80131, Naples, Italy.*

**Summary** The aim of this study was to test the hypothesis of Goldie and Coldman that the use of non-cross-resistant regimens of chemotherapy could lead to maximal anti-tumour effect. We compared standard CMF (cyclophosphamide, methotrexate, fluorouracil) with alternating CMF/EV (epirubicin, vincristine) in the adjuvant therapy of early breast cancer. Stage II premenopausal node-positive or post-menopausal node-positive oestrogen receptor-negative and stage III breast cancer patients were eligible for the study. From January 1985 to December 1990, 220 patients were randomised (115 to CMF and 105 to CMF/EV). Toxicity was mild: neurotoxicity, vomiting and hair loss were more frequent in the CMF/EV group, while permanent amenorrhoea, diarrhoea, stomachache and minor infections occurred more often in the CMF arm. At a follow-up of 48 months, 113 patients (51.4%) had had recurrence (62 on CMF and 51 on CMF/EV) and 54 (24.5%) had died (30 on CMF and 24 on CMF/EV). There was no significant difference in disease-free and overall survival between the two arms. After adjusting for menopausal status and stage, the relative risk (RR) of recurrence for CMF/EV patients was 0.93 (95% CL 0.64-1.35), while the RR of death was 0.85 (95% CL 0.49-1.47). In conclusion, the Goldie-Coldman model of alternating therapy is not confirmed in this trial of adjuvant therapy of early breast cancer, although in view of its design a difference of less than 20% in 3 year disease-free survival could not be excluded.

**Keywords:** early breast cancer; adjuvant chemotherapy; Goldie-Coldman hypothesis; alternating regimens; randomised clinical trial

Following the demonstration that adjuvant therapy, especially CMF and tamoxifen, in patients $<50$ and $\geqslant 50$ years respectively, significantly improves survival (Bonadonna *et al.*, 1976; Palshof *et al.*, 1980; NATO, 1983; Ludwig, 1984; Bianco *et al.*, 1988), efforts have been aimed at identifying patient subgroups that would benefit from the two treatments and strategies that would improve the results of treatments. A number of prospective randomised trials were designed to address specific clinical questions, e.g. optimal timing and scheduling of cytotoxic drugs and hormones; optimal duration of tamoxifen administration; and new strategies of drug administration to overcome tumour resistance.

One of the proposed models involves alternating two non-cross-resistant and equally effective drug combinations, according to the Goldie-Coldman hypothesis (Goldie *et al.*, 1982). These investigators postulated that stable genetic alterations arise in tumour cells and lead to the development of cell phenotypes characterised by drug resistance. Thus, they suggested that the early and concurrent administration of all available anti-tumour agents would be the most effective strategy. However, the overlapping toxic effects of this approach may preclude its clinical application. The next best alternative would be the use of non-cross resistant regimens which, by attacking a population of tumour cells resistant to one therapy but presumably not to the other therapy, would lead to maximal anti-tumour effect and possibly cure more patients.

At the time our study was designed, the evidence of equiactivity and non-cross-resistance of CMF (cyclophosphamide, methotrexate, 5-fluorouracil) and AV (doxorubicin, vincristine) regimens, in addition to promising results in metastatic breast cancer (De Lena *et al.*, 1975; Tormey *et al.*, 1982), provided the rationale to test the concept of the fixed rotation of the two regimens in an adjuvant setting. Furthermore, the observation that 4-epi-doxorubicin, when used alone or in combination with other cytotoxic agents, resulted in equivalent objective response rates and overall median survival in advanced breast cancer as doxorubicin parental compound-containing regimens, but with lower cardiotoxic potential (Jain *et al.*, 1985), prompted us to replace AV with EV (4-epi-doxorubicin, vincristine).

On 31 January 1985 the Cooperative Group of the University of Naples (GUN) began to recruit post-mastectomy stage II premenopausal node-positive or post-menopausal node-positive, presumably hormonoresistant, and stage III operable breast cancer patients into a single-institution randomised trial to evaluate whether adjuvant chemotherapy employing an alternating CMF/EV regimen would improve disease-free and overall survival as compared with the standard CMF regimen, and thus verify the Goldie-Coldman model of alternating regimens. In this paper we report the 9 year results of the study.

### Patients and methods

### *Patients*

Patients with histologically confirmed, unilateral breast cancer were eligible if they were: (a) stage II, premenopausal, node-positive ($N^+$) or post-menopausal, $N^+$, oestrogen receptor negative ($ER^-$); or (b) stage III. Other requirements were: age $\leqslant 75$; Karnofsky score $\geqslant 70$; normal blood counts (leucocyte $\geqslant 4\,000\ mm^{-3}$, platelet $\geqslant 100\,000\ mm^{-3}$); and normal kidney [blood urea nitrogen (BUN) and creatinine $\leqslant 1.25 \times N$] and liver [bilirubin, glutamic-oxalacetic transaminase (GOT) and alkaline phosphatase (ALP) $\leqslant 1.25 \times N$] function. Electrocardiogram and clinical examination were required to ascertain normal heart rhythm and function.

Correspondence: S De Placido
Received 27 October 1994; revised 25 January 1995; accepted 31 January 1995

Primary treatment was either radical or modified radical mastectomy or quadrantectomy for tumours ≤ 2 cm followed by high-voltage radiotherapy of the residual breast. Complete axillary node dissection was required in all patients.

Patients were considered post-menopausal if they had had their last menses at least 6 months before randomisation. Oestrogen receptor assay was requested in post-menopausal patients and performed by the biochemical assay described elsewhere (De Placido *et al.*, 1990).

Patients gave their informed consent to the study.

### *Study design and randomisation*

Within 4 weeks of surgery, eligible patients were randomly allocated to receive either six courses of CMF (control arm) or the alternating CMF/EV regimen (study arm), consisting of one course of CMF and one course of EV for a total of six courses.

Randomisation was performed by permuted blocks within strata; stratification criteria were: (a) menopausal status (pre/post), (b) stage of disease (II/III) and (c) within stage II patients the number of metastatic nodes (1–3/>3), thus creating six subgroups. Randomisation was carried out centrally by telephone at the Oncology Department's Cancer Trial Unit. The protocol design was fully approved by the Ethics Committee of the University of Naples 'Federico II'.

From 31 January 1985 to 31 December 1990, 220 patients entered the trial; 115 were assigned to standard CMF and 105 to alternating CMF/EV. The two arms were similar in the distribution of major prognostic factor (Table I), although a higher proportion of small tumours (≤ 2 cm) was observed in the CMF/EV arm.

### *Drug regimens*

The standard CMF regimen was cyclophosphamide 100 mg $m^{-2}$ orally on days 1–14, methotrexate 40 mg $m^{-2}$ and 5-fluorouracil 600 mg $m^{-2}$ intravenously on days 1 and 8 of a 28 day cycle that was repeated six times. The alternating regimen consisted of the standard 28 day CMF cycle (same dosage as the control arm) in cycles 1, 3 and 5 and a 21 day EV course of 4-epi-doxorubicin 75 mg $m^{-2}$ on day 1 and vincristine 1.4 mg $m^{-2}$ on days 1 and 8 intravenously in the even cycles, 2, 4 and 6; overall, six cycles were given, as in the control arm.

Treatment toxicity was evaluated in accordance with WHO criteria (Miller *et al.*, 1981). Amenorrhoea was defined as previously reported (Bianco *et al.*, 1991).

In both arms chemotherapy was recycled at the planned time if leucocyte and platelet counts were at least 4000 and 100 000 $mm^{-3}$ respectively. Otherwise, a 1 week delay was planned before starting the cycle. A 25% dose reduction in case of grade I toxicity and a 50% dose reduction in the case of grade II toxicity were planned on day 8 of each cycle. Day 8 was withdrawn in the case of grade III toxicity. In no case was vincristine given at a dose of more than 2.0 mg. In the case of diarrhoea and in the case of increasing liver enzymes, the dose of 5-fluorouracil and methotrexate respectively were recalculated.

Dose intensity was measured as mg $m^{-2}$ body surface area per week for each drug, regardless of the schedule used. In both the CMF and CMF/EV arms the relative dose intensity (RDI) was calculated for each patient as the ratio between delivered and planned dose intensity (Hryniuk and Bush, 1984). For these calculations, it was assumed that each of the single agents had approximately equivalent activity, and that the CMF and EV regimens were of similar efficacy.

**Table I** Characteristics of patients according to treatment arm

| *Variable* | *CMF No. (%)* | *CMF/EV No. (%)* |
|---|---|---|
| Age | | |
| ≤ 50 | 82 (71.3) | 73 (69.5) |
| > 50 | 33 (28.7) | 32 (30.5) |
| Menopausal status | | |
| Pre | 85 (73.9) | 74 (70.5) |
| Post | 30 (26.1) | 31 (29.5) |
| Stage | | |
| II | 74 (64.3) | 68 (64.8) |
| III | 41 (35.7) | 37 (35.2) |
| Tumour size (cm) | | |
| ≤ 2 | 20 (19.4) | 25 (26.0) |
| 2.1–5 | 64 (62.1) | 55 (57.3) |
| > 5 | 19 (18.5) | 16 (16.7) |
| No. of metastatic nodes | | |
| 1–3 | 60 (52.2) | 54 (51.4) |
| ≥ 4 | 55 (47.8) | 51 (48.6) |
| Histological grade | | |
| G1 + G2 | 24 (24.7) | 23 (25.3) |
| G3 | 73 (75.3) | 68 (74.7) |

### *Study parameters*

Preoperatively patients were staged with bilateral mammography, chest radiography, liver ultrasound, bone nuclear scan and segmental bone radiography in the case of positive scan. Clinical, haematological and biochemical assessment of the patients was done every 3 months for the first 2 years after surgery, every 6 months up to the fifth year and every year after the fifth. Chest radiography and liver ultrasound scan were performed every 6 months up to the fifth year and once per year from the sixth; mammography and bone nuclear scan were performed every year for 5 years and then every 2 years. Computerised axial tomography and bone X-rays were requested in the case of clinical or instrumental suspicion of disease recurrence.

### *Statistical analysis*

The present study was designed to detect a 20% difference between the two treatment arms in 3 year disease-free survival (DFS) with type I error = 0.05, type II error = 0.20 and expected 3 year DFS in the control arm = 60%. Under these conditions, about 100 patients per arm were required.

The data analysed were those available at 31, May 1994; the median follow-up was 48 months. Analyses were conducted on the basis of intention to treat. The entry date was the date of randomisation. DFS was defined as the time elapsed from randomisation to the first relapse, i.e. one of the following events: local recurrence, distant metastasis, contemporaneous local recurrence and distant metastasis, contralateral breast cancer or death without evidence of breast cancer. Overall survival (OAS) was defined as the time from randomisation to death. The Kaplan–Meier method (Kaplan and Meier, 1958) was used to estimate DFS and OAS and the Mantel–Haenszel test (Mantel, 1966) to estimate the statistical significance of the differences. The Cox proportional hazard regression model (Cox, 1972) was used for multivariate analysis where adjuvant treatment, menopausal status and stage were entered as covariates. Stage, which was defined as a three-modality variable (stage II with 1–3 positive nodes, stage II with more than three positive nodes and stage III), was coded into two dummy variables that were included in the model. Multivariate analysis results were expressed as relative risks (RR) with 95% confidence limits (95% CL). All *P*-values were two-tailed. Statistics were elaborated with the BMDP package (BMDP Statistical Software, Los Angeles, CA, USA).

### Results

### *Patient outcome*

As of 31 May 1994, 113 patients (51.4%) experienced recurrence (62 in the CMF and 51 in the CMF/EV arm) and 54 (24.5%) of them died (30 in the CMF and 24 in the CMF/EV arm). The sites of first recurrence in the treatment arms are listed in Table II. Overall there was no significant difference in DFS (Figure 1) and OAS (Figure 2) between the two arms at univariate analysis. After adjusting by stage, and nodal menopausal status, the RR of recurrence for CMF/EV treated patients was 0.93 (95% CL 0.64–1.35), while the RR of death was 0.85 (95% CL 0.49–1.47). The results of multivariate analysis are shown in Table III.

### *Side-effects*

Patients generally experienced mild toxicity, which is reported in Table IV. A higher incidence of vomiting and hair loss was observed in the CMF/EV group, while permanent amenorrhoea, diarrhoea, stomachache and minor infections were more frequent in CMF-treated patients. Peripheral neurotoxicity, i.e. constipation or paresthesies, occurred exclusively in the CMF/EV arm, as expected from vincristine toxicity.

### *Drug compliance*

In patients who received CMF, the mean relative dose intensity (RDI) for cyclophosphamide was 0.80, for methotrexate 0.81 and for 5-fluorouracil 0.83, with a mean value for the combination of 0.81. In the alternating therapy arm, the mean RDI for the individual drugs were 0.84 for cyclophosphamide, 0.89 for methotrexate, 0.89 for 5-fluorouracil, 0.79 for 4-epi-doxorubicin and 0.68 for vincristine, and the mean RDI for the treatment regimen was 0.82.

Figure 3 shows the RDI for the two combination regimens and for the single drugs in the two arms.

**Table II** Distribution of site of first relapse according to treatment arm

| *Site* | *CMF* (n = 62) | *CMF/EV* (n = 51) |
|---|---|---|
| Local | 6 (9.7) | 15 (29.4) |
| Distant | 41 (66.1) | 26 (51.0) |
| Local + distant | 5 (8.1) | 4 (7.8) |
| Second primary | 6 (9.7) | 2 (3.9) |
| Death without recurrence | 4 (6.4) | 4 (7.8) |

**Table III** Multivariate analysis for disease free and overall survival

| | *Disease-free survival* | | *Overall survival* | |
|---|---|---|---|---|
| *Variable* | *RR* | *95% CL* | *RR* | *95% CL* |
| CMF/EV *vs* CMF | 0.93 | 0.64–1.35 | 0.85 | 0.49– 1.47 |
| Stage 2 N≥4 *vs* stage 2 N1–3 | 2.27 | 1.39–3.72 | 4.54 | 1.97–10.44 |
| Stage 3 *vs* stage 2 N1–3 | 2.75 | 1.72–4.39 | 5.91 | 2.63–13.29 |
| Post- *vs* pre-menopausal | 1.01 | 0.66–1.52 | 0.90 | 0.50– 1.65 |

| No. at risk | | | | | |
|---|---|---|---|---|---|
| CMF 115 | 96 | 70 | 47 | 30 | 25 |
| CMF/EV 105 | 89 | 66 | 34 | 21 | 18 |

**Figure 1** Disease-free survival curves.

| No. at risk | | | | | |
|---|---|---|---|---|---|
| CMF 115 | 109 | 94 | 64 | 40 | 32 |
| CMF/EV | 100 | 91 | 64 | 40 | 26 |

**Figure 2** Overall survival curves.

**Table IV** Toxicity according to treatment arm

| | *CMF* *No.(%)* | *CMF/EV* *No.(%)* | P-*value* |
|---|---|---|---|
| Leucocyte (grade 2–3) | 38 (33.0) | 35 (33.3) | 0.96 |
| Haemoglobin (grade 2–3) | 21 (18.3) | 26 (24.7) | 0.24 |
| Platelet (grade 2–3) | 0 (0.0) | 4 (3.8) | 0.11 |
| Vomiting (grade 2–3) | 53 (46.1) | 61 (58.1) | 0.07 |
| Mucositis (grade 2) | 7 (6.1) | 6 (5.7) | 0.91 |
| Constipation (grade 1–2) | 10 (8.7) | 33 (31.4) | <0.0001 |
| Peripheral neurotoxicity (grade 1–2) | 0 (0.0) | 43 (41.0) | <0.0001 |
| Hair loss (grade 2–3) | 73 (63.5) | 96 (91.4) | <0.0001 |
| Amenorrhoea[a] | | | 0.72 |
| Transient | 5 (5.9) | 12 (16.2) | |
| Permanent | 58 (68.2) | 41 (55.4) | |
| Diarrhoea (grade 1–2) | 22 (19.1) | 8 (7.6) | 0.01 |
| Stomachache | 20 (17.4) | 13 (12.4) | 0.30 |
| Infections (grade 1) | 6 (5.2) | 0 (0.0) | 0.05 |
| AST/ALT (grade 1) | 7 (6.1) | 4 (3.8) | 0.44 |
| Cystitis (grade 1–2) | 9 (7.8) | 6 (5.7) | 0.53 |
| Conjunctivitis | 13 (11.3) | 8 (7.6) | 0.35 |

[a]Only for premenopausal patients.

**Figure 3** Percentiles of distribution of mean relative dose intensities (see text for calculation) for combination regimens and for single drugs within each regimen. The horizontal line of each box plot, from the upper to the lower, represents 5th, 25th, 50th, 75th and 95th percentiles of the distribution (C = cyclophosphamide, M = methotrexate, F = 5-fluorouracil, E = epi-doxorubicin, V = vincristine).

### Discussion

This study was designed to test in a clinical setting the Goldie and Coldman hypothesis that drug resistance arises before and during treatment and suggests the use of as many effective drugs as possible, as early as possible, in order to overcome the expected heterogeneity in resistance mechanisms and to maximise the probability of cure. When the overlapping toxicity prevents the simultaneous administration of all active agents, Goldie and Coldman (Goldie *et al.*, 1982) recommend that two non-cross-resistant regimens (regimen 'A' and regimen 'B') be used in a rapidly alternating fashion (i.e ABABAB) to produce optimal results. However, this strategy assumes a large degree of symmetry both (i) between the two treatment sets with respect to log kills on sensitive cells and (ii) among the cell clones with respect to mutation rates to resistance or allowable recovery times between treatment cycles.

The idea of alternating CMF and EV as a means of evaluating the Goldie and Coldman model in breast cancer was supported by the results of two clinical trials in advanced disease that strongly suggested that these regimens are equivalently active and clinically non-cross-resistant (De Lena *et al.*, 1975; Tormey *et al.*, 1982).

The results of this randomised study which compares six-cycle CMF with alternating CMF and EV schedules for a total of six cycles, failed to show a significant difference in survival between the two treatment sets. This negative result may be due to various factors.

Firstly, the survival advantage induced by CMF/EV could be very small and, thus, below the power of detection of the study.

Secondly, most of the assumptions forming the base of the Goldie-Coldman hypothesis are not met in human cancer, particularly in breast cancer. Norton and Simon (Norton and Simon, 1986; Norton, 1988), reconsidering cell growth kinetics, suggested that a single tumour is characterised by many subclones, each of them growing along a differing Gompertzian curve. Furthermore, these subclones could be not symmetrical in their resistance or in their rate of mutation toward resistance. This line of thinking seems to reflect more accurately tumour heterogeneity and the clinical situation.

Thirdly, breast cancer simply may not be a good model for the Goldie-Coldman hypothesis, because of its kinetics characteristics (slow growing tumour) and its intrinsic mechanisms of resistance (high expression of the p170 glycoprotein). In a series of randomised trials on advanced breast cancer (Kennealey *et al.*, 1978; Nemoto *et al.*, 1982; Vogel *et al.*, 1984), which varied in size from about 50 to more than 300 patients, the discouraging results were remarkably similar: alternation of regimens did not improve the response rates or survival pattern of patients with respect to those treated with a single combination regimen. One of the most interesting of these studies was that performed by the Eastern Cooperative Oncology Group (ECOG). Patients were randomised to receive either CMF or CMF alternating with AV. Interestingly, while the response rates for the rotating combination were not higher than that for the single regimen, duration of survival was significantly prolonged. However, when CMFP (CMF plus prednisone) was compared with CMFP alternating with AV, the two treatment programmes were equivalent in response rates and duration of survival, probably because CMFP is better than CMF (Tormey *et al.*, 1983).

In the adjuvant setting, two other studies have been conducted in which the use of alternating regimens was investigated to verify the mathematical model of Goldie and Coldman. A phase III trial by the ECOG randomised 533 premenopausal $N^+$ patients to receive either CMFPT (CMF plus prednisone and tamoxifen) or the same regimen plus halotestin alternating monthly with VATHT (vinblastine, doxorubicin, thiotepa, halotestin, tamoxifen). At a 5.1 year follow-up, the time to relapse was superior for the alternating regimen, while there was no statistical difference between the two treatment regimens in terms of overall survival (Tormey *et al.*, 1992). Another prospective randomised trial examined the effect of CMF *vs* alternating CMF/AV in stage II, $N^+$ patients. At 4 years there was no difference in outcome between the treatment arms (Chaitchik *et al.*, 1989).

The Goldie and Coldman alternating strategy has also been clinically tested in Hodgkins's disease and in small-cell lung cancer (SCLC). These malignancies seem to be useful models, being among the few cancers for which many effective drug treatments are available. Although an early trial in Hodgkin's disease suggested a significant prolongation in DFS and OS (Bonadonna *et al.*, 1986), subsequent studies did not find that alternating regimens provided significantly better results than did adequately used four-drug combinations (Longo *et al.*, 1991; Canellos *et al.*, 1992). Recently, at least five large randomised trials have tested the use of alternating non-cross resistant chemotherapy in SCLC. Of the four studies conducted in patients with extensive-stage disease (Evans *et al.*, 1987; Fukuoka *et al.*, 1991; Wolf *et al.*, 1991; Roth *et al.*, 1992), only one trial found a significant improvement of median survival in favour of the alternating schedule, but the magnitude of benefit was modest (Evans *et al.*, 1987). Similarly, for patients with limited-stage disease, the median survival was significantly better with the alternating regimen in one (Fukuoka *et al.*, 1991) of the three reported studies (Goodman *et al.*, 1990; Fukuoka *et al.*, 1991; Wolf *et al.*, 1991). However, this positive result must be interpreted with caution, given the small number of limited-stage patients included in the trial.

In conclusion, in breast cancer, as well as in other malignancies, the data available do not support the hypothesis of a significant advantage for the use of rapidly alternating schedules as compared with a single active regimen. In addition, toxicity is often more pronounced with alternating regimens.

**Acknowledgements**
This study was funded by the National Research Council (CNR) with Grants Nos. 86.00530.44, 87.01188.44 and 88.00530.44. Chiara Carlomagno is a recipient of a fellowship awarded by the Italian Association for Cancer Research (AIRC).

### References

BIANCO AR. DE PLACIDO S. GALLO C. PAGLIARULO C. MARINELLI A. PETRELLA G. D'ISTRIA M AND DELRIO G. (1988). Adjuvant therapy with tamoxifen in operable breast cancer. 10 year results of the Naples (GUN) study. *Lancet*. **ii**, 1095–1099.

BIANCO AR. DEL MASTRO L. GALLO C. PERRONE F. MATANO E. PAGLIARULO C AND DE PLACIDO S. (1991). Prognostic role of amenorrhea induced by adjuvant chemotherapy in premenopausal patients with early breast cancer. *Br. J. Cancer*. **63**, 799–803.

BONADONNA G. BRUSAMOLINO E. VALAGUSSA P. ROSSI A. BROGNATELLI L. BRAMBILLA C. DE LENA M. TANCINI G. BAJETTA E. MUSUMECI R AND VERONESI U. (1976). Combination chemotherapy as an adjuvant treatment in operable breast cancer. *N. Engl. J. Med*.. **294**, 405–410.

BONADONNA G. VALAGUSSA P AND SANTORO A. (1986). Alternating non-cross-resistant combination chemotherapy or MOPP in stage IV Hodgkin's disease. *Ann. Intern. Med*.. **104**, 730–746.

CANELLOS GP. ANDERSON JR. PROPERT KJ. NISSEN N. COOPER MR. HENDERSON ES. GREEN MR. GOTTLIEB A AND PETERSON BA. (1992). Chemotherapy of advanced Hodgkin's disease with MOPP. ABVD. or MOPP alternating with ABVD. *N. Engl. J. Med*.. **327**, 1478–1484.

CHAITCHIK S. BOROVIK R. ROBINSON E. PALTI S. BIRAN S. BRUFMAN G. BRENER H. RATH P AND SPICER E. (1989). Adjuvant chemotherapy for stage II breast cancer: CMF *vs* alternating CMF-VA. A national randomized trial. *Proc. Am. Soc. Clin. Oncol*.. **8**, 186.

COX DR. (1972). Regression models and life table. *J.R. Stat. Soc. B*. **34**, 187–220.

DE LENA M. BRAMBILLA C. MORABITO A AND BONADONNA G. (1975). Adriamycin plus vincristine compared to and combined with cyclophosphamide. methotrexate. and 5-fluorouracil for advanced cancer. *Cancer*. **35**, 1108–1115.

DE PLACIDO S. GALLO C. MARINELLI A. PERRONE F. PAGLIARULO C. PETRELLA G. DELRIO G. D'ISTRIA M. DEL MASTRO L AND BIANCO AR. (1990). Relationship between steroid hormone receptor levels and adjuvant tamoxifen in early breast cancer. Ten-year results of the Naples (GUN) study. *Breast Cancer Res. Treat*.. **16**, 111–117.

EVANS WK. FELD R. MURRAY N. WILLAN A. COY P. OSOBA D. SHEPHERD FA. CLARK DA. LEVITT M. MACDONALD A. WILSON K. SHELLEY W AND PATER J. (1987). Superiority of alternating non-cross-resistant chemotherapy in extensive small cell lung cancer. *Ann. Intern. Med*.. **107**, 451–458.

FUKUOKA M. FURUSE K. SAIJO N. NISHIWAKI Y. IKEGAMI H. TAMURA T. SHIMOYAMA M AND SUEMASU K. (1991). Randomized trial of cyclophosphamide. doxorubicin. and vincristine versus cisplatin and etoposide versus alternation of these regimens in small-cell lung cancer. *J. Natl Cancer Inst*.. **83**, 855–861.

GOLDIE JH. COLDMAN AJ AND GRUDAUSKAS GA. (1982). Rationale for the use of alternating non cross resistant chemotherapy. *Cancer Treat. Rep*.. **66**, 439–449.

GOODMAN GE. CROWLEY JJ. BLASKO JC. LIVINGSTON RB. BECK TM. DEMATTIA MD AND BUKOWSKI RM. (1990). Treatment of limited small-cell lung cancer with etoposide and cisplatin alternating with vincristine. doxorubicin and cyclophosphamide versus concurrent etoposide. vincristine. doxorubicin. and cyclophosphamide and chest radiotherapy: a Southwest Oncology Group study. *J. Clin. Oncol*.. **8**, 39–47.

HRYNIUK WM AND BUSH H. (1984). The importance of dose intensity in chemotherapy of metastatic breast cancer. *J. Clin. Oncol*.. **2**, 1281–1288.

JAIN KK. CASPER ES. GELLER NL. HAKES TB. KAUFMAN RJ. CURRIE V. SCHWARTZ W. CASSIDY C. PETRONI GR. YOUNG CW AND WITTES RE. (1985). A prospective randomized comparison of epirubicin and doxorubicin in patients with advanced breast cancer. *J. Clin. Oncol*.. **3**, 818–826.

KAPLAN EL. MEIER P. (1958). Non parametric estimation for incomplete observation. *J. Am. Stat. Assoc*.. **53**, 457–481.

KENNEALEY GT. BOSTON B. MITCHELL MS. KNOBF MK. BOBROW SN. PEZZIMENTI JF. LAWRENCE R. BERTINO JR. (1978). Combination chemotherapy for advanced breast cancer. *Cancer*. **42**, 27–33.

LONGO DL. DUFFEY PL. DE VITA VT. WIERNIK PH. HUBBARD SM. PHARES JC. BASTIAN AW. JAFFE ES. YOUNG RC. (1991). Treatment of advanced-stage Hodgkin's disease: alternating non-cross-resistant MOPP CABS is not superior to MOPP. *J. Clin. Oncol*.. **9**, 1409–1420.

LUDWIG BREAST CANCER STUDY GROUP. (1984). Randomised trial of chemo-endocrine therapy. endocrine therapy. and mastectomy alone in postmenopausal patients with operable breast cancer and axillary nodal metastases. *Lancet*. **i**, 1253–1256.

MANTEL N. (1966). Evaluation of survival data and two new rank order statistics arising in its consideration. *Cancer Chemother. Rep*.. **50**, 163–170.

MILLER AB. HOOGSTRATEN B. STAQUET M. WINKLER A. (1981). Reporting results of cancer treatment. *Cancer*. **47**, 207–214.

NEMOTO T. HORTON J. SIMON R. DAO TL. ROSNER D. CUNNINGHAM T. SPONZO R. SNYDERMAN M. (1982). Comparison of four combination chemotherapy programs in metastatic breast cancer. *Cancer*. **49**, 1988–1993.

NOLVADEX ADJUVANT TRIAL ORGANISATION. (1983). Controlled trial of tamoxifen as adjuvant agent in management of early breast cancer. *Lancet*. **i**, 257–261.

NORTON L. (1988). A Gompertzian model of human breast cancer growth. *Cancer Res*.. **48**, 7067–7071.

NORTON L. SIMON R. (1986). The Norton-Simon hypothesis revisited. *Cancer Treat. Rep*.. **70**, 163–169.

PALSHOF T. MOURIDSEN HT. DAEHNFELDT JL. (1980). Adjuvant endocrine therapy of primary breast cancer. Report on the Copenhagen breast cancer trials. *Eur. J. Cancer*. **2**, (Suppl.). 183–187.

ROTH BJ. JOHNSON DH. EINHORN LH. SCHACTER LP. CHERNG NC. COHEN HJ. CRAWFORD J. RANDOLPH JA. GOODLOW JL. BROUN GO. OMURA GA. GRECO FA. (1992). Randomized study of cyclophosphamide. doxorubicin. and vincristine versus etoposide and cisplatin versus alternation of these two regimens in extensive small-cell lung cancer: a phase III trial of the Southeastern Cancer Study Group. *J. Clin. Oncol*. **10**, 282–291.

TORMEY DC. GELMAN R. BAND PR. SEARS M. ROSENTHAL SN. DEWYS W. PERLIA C. RICE MA. (1982). Comparison of induction chemotherapies for metastatic breast cancer. *Cancer*. **50**, 1235–1244.

TORMEY DC. GELMAN R. FALKSON G. (1983). Prospective evaluation of rotating chemotherapy in advanced breast cancer. *Am. J. Clin. Oncol*.. **6**, 1–18.

TORMEY DC. GRAY R. ABELOFF MD. ROSEMAN DL. GILCHRIST KW. BARYLAK EJ. STOTT P. FALKSON G. (1992). Adjuvant therapy with a doxorubicin regimen and long-term tamoxifen in premenopausal breast cancer patients: an Eastern Cooperative Oncology Group trial. *J. Clin. Oncol*.. **10**, 1848–1856.

VOGEL CL. SMALLEY RV. RANEY M. KRAUSS S. CARPENTER J. VELEZ-GARCIA E. FISHKIN E. RAAB S. MOORE MR. STAGG M. (1984). Randomized trial of cyclophosphamide. doxorubicin. and 5-fluorouracil alone or alternating with a 'cycle active' non-cross-resistant combination in women with visceral metastatic breast cancer: a Southeastern Cancer Study Group Project. *J. Clin. Oncol*.. **2**, 643–651.

WOLF M. PRITSCH M. DRINGS P. HANS K. SCHROEDER M. FLECHTNER H. HEIM M. HRUSKA D. MENDE S. BECKER H. DANNHAUSER J. LOHMULLER R. GROPP C. GASSEL WD. HOLLE R AND HAVEMANN K. (1991). Cyclic-alternating versus response-oriented chemotherapy in small-cell lung cancer. A German multicenter randomized trial of 321 patients. *J. Clin. Oncol*.. **9**, 614–624.